# Wedding Rings

**Published:** 1891-12

**Authors:** 


### WEDDING RINGS.

You want to know why the ring is used as a matrimonial pledge? said a learned Smithsonian curator to a *Washington Star* writer. The reason is very well known. It is employed as a token of good faith because the ring was originally and primarily a seal. In ancient Babylon, 4,000 years ago, all documents were attested by seals, as they are now, and the merchants very usually wore their seals on their finger-rings of gold and other metals. With these signets they impressed their own private and particular devices upon the agreements and contracts of all sorts, thus making them good and binding. Documents in those days were not written upon paper, but with a wooden stylus upon moist clay, which was subsequently hardened by baking.

From this source the seal has come down through the more modern civilization of Greece and Rome to the present day as a sign of good faith. It is with that significance that it is placed upon the third finger of the bride's left hand to seal the contract between herself and her newly made husband. Also it is surmised that the ring is intended to remind the wife of the fidelity she owes. Furthermore, the circle is the emblem of eternity. But why, you ask, does the bride choose the third finger of the left hand to wear her ring on? Simply because the ancients supposed that a nerve ran directly from that finger to the heart. I need hardly say that the researches of modern anatomists have shown this to be an error; but the custom survives. The courtesans of Rome used to wear their rings upon their thumbs because the thumb was sacred to Venus.

Hebrews regard the ring in the ceremony of marriage as of extraordinary importance. It must be of a certain value, certified to by the officiating rabbi, and it must be absolutely the groom's own property—not obtained by gift or purchased on credit. There are a number of curious superstitions about wedding rings. If one is broken it signifies to the wife that she is going to lose her husband. I really think that a majority of women never take off their wedding rings, believing that to do so would occasion misfortune.—*Ex.*

